# The epidemiology of cephalosporin allergy labels in pediatric primary care

**DOI:** 10.1017/ash.2023.487

**Published:** 2023-11-29

**Authors:** Torsten Joerger, Margaret G. Taylor, Debra L. Palazzi, Jeffrey S. Gerber

**Affiliations:** 1 Department of Pediatrics, Division of Infectious Diseases, University of Pennsylvania Perelman School of Medicine and Children’s Hospital of Philadelphia, Philadelphia, PA, USA; 2 Department of Pediatrics, Division of Infectious Diseases, Baylor College of Medicine and Texas Children’s Hospital, Houston, TX, USA

## Abstract

**Background:**

Recent studies have sought to understand the epidemiology and impact of beta-lactam allergy labels on children; however, most of these studies have focused on penicillin allergy labels. Fewer studies assess cephalosporin antibiotic allergy labels in children. The objective of this study was to determine the prevalence, factors associated with, and impact of cephalosporin allergy labels in children cared for in the primary care setting.

**Methods:**

Cephalosporin allergy labels were reviewed among children in a dual center, retrospective, birth cohort who were born between 2010 and 2020 and followed in 90 pediatric primary care practices. Antibiotic prescriptions for acute otitis media were compared in children with and without cephalosporin allergies.

**Results:**

334,465 children comprised the birth cohort and 2,877 (0.9%) were labeled as cephalosporin allergic during the study period at a median age of 1.6 years. Third-generation cephalosporins were the most common class of cephalosporin allergy (83.0%). Cephalosporin allergy labels were more common in children with penicillin allergy labels than those without (5.8% vs. 0.6%). Other factors associated with a cephalosporin allergy label included white race, private insurance, presence of a chronic condition, and increased health care utilization. Children with third-generation cephalosporin allergy labels received more amoxicillin/clavulanate (28.8% vs. 10.2%) and macrolides (10.4% vs. 1.9%) and less amoxicillin (55.8% vs. 70.9%) for treatment of acute otitis media than non-allergic peers *p* < 0.001.

**Conclusions:**

One in 100 children is labeled as cephalosporin allergic, and these children receive different antibiotics for the treatment of acute otitis media compared to non-allergic peers.

## Introduction

Beta-lactam antibiotics include penicillins, cephalosporins, monobactams, and carbapenems, and are the most commonly prescribed antibiotic class in the United States.^1^ Hypersensitivity reactions to drugs are documented in nearly 10% of patients, and beta-lactam antibiotics are the most commonly implicated drugs.^2^ Among beta-lactam antibiotics, most research has focused on penicillin allergies, demonstrating that five to ten percent of children carry a penicillin allergy label but that most children with these labels do not have type 1 IgE-mediated hypersensitivities on formal evaluation and tolerate penicillin antibiotics after an allergy label is placed.^3–6^ Reasons for mislabeling may include anticipated side effects of drugs (ex. diarrhea) and non-allergic cutaneous reactions such as viral exanthems.^4^ Substantial harms to carrying erroneous penicillin allergy labels, mediated through the use of alternative antibiotics, include increased adverse events, prolonged hospital stays, *Clostridioides difficile* infection, acute kidney injury, and antimicrobial resistance.^7,8^ After penicillins, cephalosporins are the second most commonly prescribed antibiotics with over 30 million outpatient prescriptions in the United States in 2021.^9^ Despite their frequent use, little is known about the epidemiology of cephalosporin allergy labels in children, particularly in the outpatient setting where most antibiotics are prescribed.

This study aimed to describe the epidemiology and factors associated with cephalosporin allergy labels among children in two large pediatric primary care networks. In addition, we describe the impact of having a cephalosporin allergy on antibiotic prescriptions among children treated for acute otitis media (AOM), the most common indication for antibiotic prescribing in children.

## Methods

### Study design and setting

This was a retrospective birth cohort study conducted in two pediatric primary care networks: the Children’s Hospital of Philadelphia Care Network (CHOP) and the Texas Children’s Primary Care Network (TCP). These two networks encompass 90 associated pediatric primary care clinics that care for more than 700,000 children with more than 2 million encounters annually. To the authors' knowledge, there were no formal quality improvement initiatives to address beta-lactam allergy de-labeling in either setting during the study period. The CHOP and Baylor College of Medicine Institutional Review Boards for the Protection of Human Subjects approved this study.

### Study population

Children in the CHOP and TCP primary care networks were included in the birth cohort if they met the following criteria: (1) born between January 1st 2010 and June 30th 2020, (2) seen by a pediatrician within the first 2 weeks of life, and (3) had a minimum of two additional primary care visits by their first birthday. Qualifying visits could be either well child or acute care visits. To study a group of children who were likely to get most of their care within these two primary care networks, children were censored if they did not have a minimum of one primary care encounter per year in the first four years of life and at least one primary care encounter every two years thereafter. Children were censored from the birth cohort on the date of their last qualifying primary care visit.

### Variables/outcomes

Both primary care networks use the EpicCare (Epic Systems, Inc; Verona, WI) Electronic Health Record (EHR) from which demographic variables including sex, race/ethnicity, and date of birth were abstracted. Race and ethnicity data are self-reported into the EHR and were subsequently classified into the following groups: non-Hispanic White, non-Hispanic Black, Hispanic, Asian or Pacific Islander, or other, which included races with low representation in this cohort such as American Indian and Alaskan Native as well as self-reported other. In addition, for each healthcare encounter, the International Classification of Diseases, 10^th^ edition (ICD-10) code(s) assigned, the location of the clinic, insurance payer, and medications prescribed were obtained. Subjects with at least one encounter with government insurance were classified as having government insurance, with the remainder of children classified as having private insurance. A subject was classified as having a chronic condition if their problem list included a code for one or more conditions defined in the pediatric complex chronic conditions system version 2 at any time during their inclusion in the birth cohort.^10^ Subjects’ problem lists and data from the allergy tab were recorded. The data were extracted on November 12, 2020 for TCP clinics and on December 13, 2020 for CHOP clinics. The validity of the electronically extracted data was confirmed by manual chart review of a subset of subjects from each of the health systems and continued until no errors were identified.

The exposure of interest was the presence of a cephalosporin allergy label, defined as documentation in the EHR allergy tab of an allergy to any cephalosporin and further classified as first, second, third, fourth, or unspecified generation of cephalosporin. Penicillin allergy labels were separately classified as previously defined.^5,7^ The prevalence of cephalosporin allergy labels, the timing of their placement in the EHR, factors associated with their placement, and the timing of any associated penicillin allergy labels were assessed. Cephalosporin allergy labels that were later removed during the study period were classified as de-labeled. Additionally, as AOM is the most common indication for antibiotic prescribing in the pediatric primary care setting, we assessed antibiotics used to treat AOM in children with penicillin allergies, third-generation cephalosporin allergies, or both, and compared these to children without a penicillin or cephalosporin allergy. Acute otitis media was defined by ICD-10 codes H65-H67. For the analysis, encounters where an antibiotic was prescribed within the previous 30 days were excluded, as recent prescriptions may have influenced antibiotic choice. In addition, subjects with a concurrent ICD-10 code for another infection (ex. sinusitis) were excluded from the analysis.

### Statistical methods

Descriptive statistics were expressed as frequencies or as medians with interquartile ranges for categorical and continuous variables, respectively. A logistic regression model was used to assess for variables associated with a cephalosporin allergy label. These variables were selected a priori based on clinical knowledge and previous research in children with other antibiotic allergies and included: sex, race/ethnicity, presence of a chronic condition, private or public insurance, and number of health care visits in the first two years of life.^5^ Presence of penicillin allergy label was not incorporated into the regression model because in children, eventually labeled as both cephalosporin and penicillin allergic, the penicillin allergy does not always precede the cephalosporin allergy label. A chi square test was used to determine differences in the frequency of antibiotic prescribing based on allergy label status. A two-sided 5% significance level (*P* < 0.05) was used for all statistical inferences, and all analyses were conducted using Stata software 16 (StataCorp).

## Results

### Epidemiology of cephalosporin allergy labels

In total, 334,465 children with over 1.4-million-person years of healthcare encounters were included in the birth cohort. Additional demographic information on the birth cohort has previously been reported.^5^ A total of 2,877 children (0.9%) were labeled as cephalosporin allergic during the study period, compared to 18,015 (5.4%) labeled as penicillin allergic. The prevalence of children labeled as cephalosporin allergic varied between 0.13% and 2.7% among the 90 primary care clinics assessed. Children who identified as White race, used private insurance, had a chronic condition, or had a penicillin allergy were most likely to acquire a cephalosporin allergy label (Table 1). Most (2,387; 83.0%) cephalosporin allergies were for third-generation cephalosporins, followed by first generation (226; 7.9%), second generation (107; 3.7%) and fourth generation (1; 0.03%). A total of 197 (6.8%) children carried a label of cephalosporin allergy without defining a specific drug or generation, and 60 (2.1%) children had multiple cephalosporins listed in the allergy tab. Among the 2,877 children with a cephalosporin allergy, 1,043 (36.2%) also had a penicillin allergy label.

**Table 1.** Demographics of children with a cephalosporin allergy label in birth cohort

Characteristic	Total cohort *n* = 334,465	Cephalosporin allergy *n* = 2,877 (0.9%)
**Sex**		
Female	164,173 (49.1%)	1,390 (0.8%)
Male	170,292 (50.9%)	1,487 (0.9%)
**Race and ethnicity**		
Non-Hispanic White	148,534 (44.4%)	1,777 (1.2%)
Hispanic	72,831 (21.8%)	574 (0.8%)
Non-Hispanic Black	59,598 (17.8%)	200 (0.3%)
Non-Hispanic Asian or Pacific Islander	21,081 (6.3%)	139 (0.7%)
Other	13,746 (4.1%)	104 (0.8%)
Missing	18,675 (5.6%)	83 (0.4%)
**Payer**		
Public	119,988 (35.9%)	659 (0.6%)
Private	214,477 (64.1%)	2,218 (1.0%)
**Chronic condition**		
Yes	24,571 (7.4%)	276 (1.1%)
No	309,894 (92.6%)	2,601 (0.8%)
**Penicillin allergy**		
Yes	18,015 (5.4%)	1,043 (5.8%)
No	316,450 (94.6%)	1,834 (0.6%)

**Figure 1.**
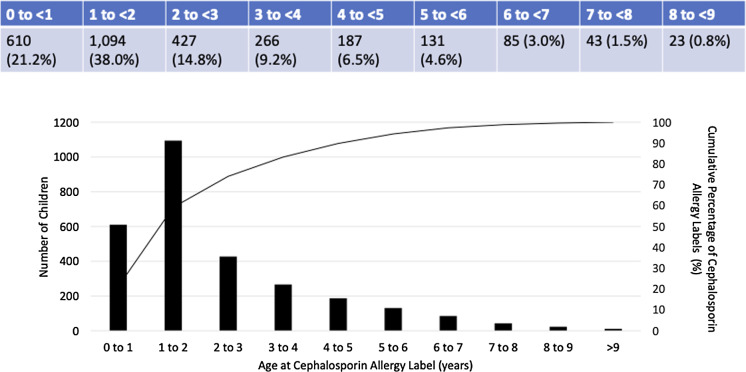
Proportion of children labeled as cephalosporin allergic by age.

The median age and interquartile range of acquiring a cephalosporin allergy label were 1.6 [1.0–3.0] years, but children labeled as allergic to first-generation cephalosporins were labeled older (2.6 [1.2–4.6] years) as compared to second (1.7 [1.1–2.9] years) and third-generation cephalosporins (1.6 [1.0–3.0] years). More than 20% of cephalosporin allergy labels were acquired in the first year of life and nearly all by four years of age (Figure 1). Among children with both penicillin and cephalosporin allergy labels, the penicillin allergy label was acquired first most (73.9%) of the time and occurred a median of 0.76 [0.21–2.0] years before the acquisition of a cephalosporin allergy label.

Of the 2,877 children with a cephalosporin allergy label, 312 (11%) were de-labeled during the study period, at a median of 1.21 [0.36–2.39] years after cephalosporin allergy label placement. Children with both penicillin and cephalosporin allergy labels [149 of 1,043 (14.3%)] were more likely than those with only a cephalosporin allergy label [163 of 1,834 (8.9%)] to have their cephalosporin allergy removed (*p*-value <0.001). Race, ethnicity, sex, and insurance status did not impact cephalosporin allergy de-labeling.

### Factors associated with cephalosporin allergy labels

Cephalosporin allergy occurred more commonly in children with a penicillin allergy label than those without (5.8% vs 0.6%). In a multivariable logistic regression model, after adjusting for total time in the birth cohort, Non-Hispanic Black children (aOR 0.51 [95%CI 0.44–0.60]) and, to a lesser extent, non-Hispanic Asian or Pacific Islander (aOR 0.72 [95%CI 0.61–0.87]) were less likely than White children to have received a cephalosporin allergy label. Children with government insurance were significantly less likely to acquire a cephalosporin allergy label (aOR 0.74 [95%CI 0.67–0.81]). The presence of a chronic condition increased the likelihood of a cephalosporin allergy label (aOR 1.24 [95%CI 1.09–1.41]) as did increased healthcare utilization with each additional healthcare visit before two years of age increasing the risk of cephalosporin allergy labeling by 10% (Table 2).

**Table 2.** Univariable and multivariable logistic regression model for cephalosporin allergy labeling

Characteristic	Univariable analysis OR (95% CI)	*P*-value	Multivariable analysis OR (95% CI)*	*P*-value
**Sex**				
Female	1 [Reference]	NA	1 [Reference]	NA
Male	1.03 [0.96–1.11]	0.406	0.92 [0.86–0.99]	0.039
**Ethnicity and race**				
Non-Hispanic White	1 [Reference]	NA	1 [Reference]	NA
Hispanic	0.66 [0.60–0.72]	<0.001	0.91 [0.82–1.01]	0.07
Non-Hispanic Black	0.28 [0.24–0.32]	<0.001	0.51 [0.44–0.60]	<0.001
Non-Hispanic Asian or Pacific Islander	0.55 [0.46–0.65]	<0.001	0.72 [0.61–0.87]	<0.001
**Payer**				
Private Insurance	1 [Reference]	NA	1 [Reference]	NA
Government Insurance	0.52 [0.48–0.58]	<0.001	0.74 [0.67–0.81]	<0.001
**Chronic Condition**	1.34 [1.18–1.52]	<0.001	1.24 [1.09–1.41]	0.001
**Healthcare Visits by 2 years of Age**	1.11 [1.10–1.11]	<0.001	1.10 [1.10–1.11]	<0.001

* Additionally adjusted for years in the birth cohort.

### Antibiotic prescribing for acute otitis media among children with cephalosporin allergy labels

**Figure 2.**
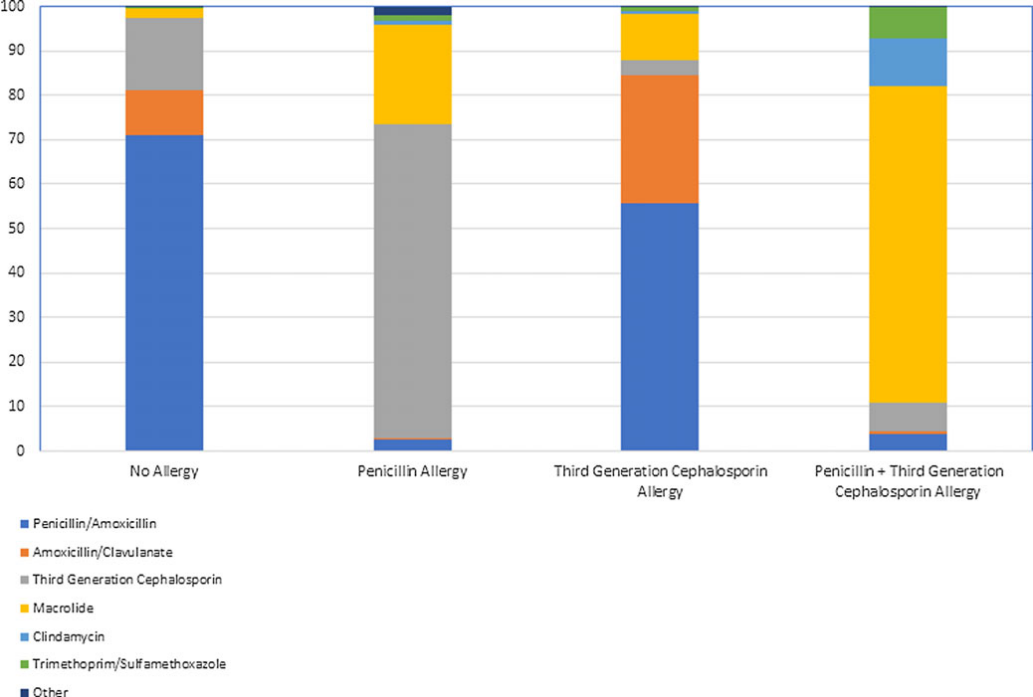
Distribution of antibiotics prescribed for acute otitis media by allergy status.

Among 526,710 episodes of AOM that occurred in 188,393 children who had not received an antibiotic in the preceding 30 days, children with isolated third-generation cephalosporin allergy labels received more amoxicillin/clavulanate (28.8% vs. 10.2%) and less amoxicillin (55.8 % vs. 70.9%) than children without a third-generation cephalosporin allergy (*p* < 0.001, Figure 2). Macrolides were prescribed to 10% of children with a third-generation cephalosporin allergy, 22% of children with a penicillin allergy, and over 70% of children with both a penicillin and third-generation cephalosporin allergy. Clindamycin and trimethoprim/sulfamethoxazole prescribing was rare, except for children with both a penicillin and cephalosporin allergy at the time of their infection.

## Discussion

In this retrospective birth cohort study of over 360,000 children from 90 pediatric primary care practices, 0.9% of children were labeled as cephalosporin allergic, most commonly due to third-generation cephalosporin allergies. Children were labeled young, and those with a penicillin allergy label were more likely to have a cephalosporin allergy label. Children with cephalosporin allergy labels were prescribed different antibiotics for treatment of AOM than non-allergic peers.

The prevalence of cephalosporin allergy labels in this study is similar to prior studies and lower than the prevalence of penicillin allergy labels among children in this cohort.^3,5^ The difference in allergy labeling rates between penicillins and cephalosporins may be related to a differential propensity to cause hypersensitivity reactions among beta-lactams with differing R_1_ side chains or lower rates of cephalosporin antibiotic prescribing in primary care settings.^9,11,12^ Additionally, when a child develops a rash or other adverse reaction, it is possible that pediatricians may be less likely to label a child as allergic when a cephalosporin is prescribed and place more scrutiny on a reaction given a perceived lack of alternative antibiotics to treat subsequent infections. Our study suggests that future research examining beta-lactam antibiotic allergies should distinguish between penicillin and cephalosporin allergies and avoid combining these two drug classes to better understand the differences between them.

Children with a penicillin allergy label were more likely to be labeled as cephalosporin allergic than those without a penicillin allergy label. One potential explanation for this finding is that children with a penicillin allergy label are more likely to be prescribed a cephalosporin than their non-allergic peers, which may increase the future risk of obtaining a cephalosporin allergy label.^7^ In addition, since most patients labeled as penicillin allergic can tolerate penicillin antibiotics, and only 2% of children with a true penicillin allergy label are expected to have cross re-activity with cephalosporins, these dual labeled patients could represent a group of children that are more prone to viral and/or drug-mediated non-IgE rashes, or more likely to seek care for adverse events than children without these allergy labels.^4,6^ Children were also more likely to acquire a cephalosporin allergy label if they were White, had private insurance, had a complex medical condition, or increased healthcare utilization, similar to the risk factors for acquiring a penicillin allergy label.^5^ The reasons behind these racial disparities are unclear but may include differential antibiotic prescribing practices among health care providers and unequal access to healthcare.^13–15^

As with children labeled as penicillin allergic, children labeled as cephalosporin allergic were infrequently de-labeled in this study. A recent study examining reported cephalosporin allergies in children seen in a pediatric emergency department found that nearly 60% had reaction histories that were low-risk for being IgE or T-cell mediated processes, indicating that a large proportion of children labeled as cephalosporin allergic would be good candidates for drug challenge and potential de-labeling.^16^ Reasons behind low rates of cephalosporin allergy de-labeling are not clear but may include: less emphasis on cephalosporin allergies and less awareness of consequences of cephalosporin allergy labels as compared to penicillin allergy labels. Future research should determine reasons for low rates of cephalosporin allergy de-labeling and identify ways to increase rates of de-labeling. Lastly, children with both penicillin and cephalosporin allergy labels were more likely to be de-labeled than children with only cephalosporin allergy labels. We hypothesize that physicians are more likely to refer to an allergist or otherwise evaluate an allergy label when they run out of antibiotic options. This hypothesis should be further evaluated.

The American Academy of Pediatrics recommends amoxicillin as first line for AOM, except if the child has taken amoxicillin in the previous 30 days, if concurrent conjunctivitis is noted, or if the child is allergic to penicillin.^17^ We hypothesized that children with third-generation cephalosporin allergies would be prescribed amoxicillin at the same rate as children without beta-lactam allergies given the limited cross-reactivity between third-generation cephalosporins and amoxicillin.^18,19^ However, we found that nearly 30% of patients with a third-generation cephalosporin allergy were given amoxicillin/clavulanate instead of amoxicillin. Some children may have had concurrent conjunctivitis, and we cannot exclude that some patients failed amoxicillin prescribed at a clinic outside of the CHOP or TCP health systems, but we suspect these to be uncommon in this birth cohort and similar between the third-generation cephalosporin allergic and non-allergic groups. Physicians frequently prescribe broader spectrum antimicrobial agents than are needed, despite lack of benefits and increased adverse events.^20^ Despite suboptimal antibiotic susceptibility rates and American Academy of Pediatrics guideline recommendations advising against using macrolides for AOM, macrolides were prescribed in 10% of children with a cefdinir allergy, 22% with a penicillin allergy, and 71% of children with both allergy labels.^17,21^ Given the limited efficacy of macrolides against typical AOM pathogens, children with cephalosporin allergies should be referred to allergy specialists for formal evaluation in order to optimize future prescribing.

Assuming a pediatric population of 80 million, and a prevalence of 0.9%, it can be estimated that more than 700,000 children in the United States have a cephalosporin allergy label.^22^ The presence of a penicillin allergy increases the rate of broad-spectrum antibiotic prescribing and antibiotic-related costs in children hospitalized with pneumonia.^23^ In adults, beta-lactam allergies also increase the rate of bacteremia treatment failure and rates of adverse events such as acute kidney injury and *Clostridioides difficile* infection.^24–26^ Future studies should examine the impact of cephalosporin allergy labels on children treated for outpatient infections.

The strengths of this study include its large sample size of over 300,000 children cared for in 90 primary care clinics across three states and two health systems. These patients represent a racially, socioeconomically, and geographically diverse population, which increases generalizability. Limitations of this study include its dependance on EHR data; therefore, factors that may have influenced care decisions that were not included in the variables examined cannot be accounted for. In addition, we cannot exclude that some patients received care outside of the CHOP or TCP systems, however, the birth cohort design with strict censoring criteria likely minimized this limitation.

## Conclusion

In this study of over 300,000 primary care patients, 0.9% were labeled as cephalosporin allergic. White children, those with private insurance, chronic conditions, increased healthcare utilization, and penicillin allergy labels, were associated with cephalosporin allergy labels. Children with cephalosporin allergy labels received different, often more broad, and potentially suboptimal antibiotics for the treatment of acute otitis media. Future studies should evaluate the safety of cephalosporin allergy de-labeling to optimize antibiotic prescribing in the pediatric outpatient setting.
